# Resource Allocation for a Secure SWIPT Network Based on a Quantitative Energy Harvesting Mechanism

**DOI:** 10.3390/s23115117

**Published:** 2023-05-27

**Authors:** Long Zhu, Liang Xue, Xuan Gong, Chunjie Wang

**Affiliations:** 1 School of Information and Electrical Engineering, Hebei University of Engineering, Handan 056038, China; 2 Institute of Advanced Computing and Digital Engineering, Shenzhen Institute of Advanced Technology, Chinese Academy of Sciences, Shenzhen 518055, China

**Keywords:** energy harvesting, artificial noise, power mapping table, QPS receiver, simultaneous wireless information and power transfer

## Abstract

Simultaneous wireless information and power transfer (SWIPT) technology can effectively extend the lifecycle of energy-constrained networks. In order to improve the energy harvesting (EH) efficiency and network performance in secure SWIPT networks, this paper studies the resource allocation problem based on the quantitative EH mechanism in the secure SWIPT network. Based on a quantitative EH mechanism and nonlinear EH model, a quantified power-splitting (QPS) receiver architecture is designed. This architecture is applied in the multiuser multi-input single-output secure SWIPT network. With the goal of maximizing the network throughput, the optimization problem model is established under the conditions of meeting the legal user’s signal-to-interference-plus-noise ratio (SINR), EH requirements, the total transmit power of the base station, and the security SINR threshold constraints. Due to the coupling of variables, the problem is a nonconvex optimization problem. To deal with the nonconvex optimization problem, a hierarchical optimization method is adopted. Firstly, an optimization algorithm based on the optimal received power of EH circuit is proposed, and a power mapping table is constructed through the optimization algorithm, from which the optimal power ratio to meet the user’s EH requirements is obtained; then, the nonconvex problem is transformed into a convex problem by using variable substitution, semidefinite relaxation, dichotomous optimization, etc. The simulation results show that compared with the power splitting receiver architecture, the input power threshold range of the QPS receiver architecture is larger, which can avoid the EH circuit falling into the saturated working area and maintain high network throughput.

## 1. Introduction

With the large-scale commercial use of 5G technology, the situation of the Internet of Everything has gradually formed [1,2,3], energy-constrained networks such as wireless sensor networks and remote wireless LANs are troubled by the continuous supply of energy, and it is difficult to achieve large-scale development and application [4,5]. Simultaneous wireless information and power transfer (SWIPT) technology uses radio frequency (RF) signals to carry information and energy and simultaneously realizes information decoding (ID) and energy harvesting (EH) [6,7,8]; it prolongs the lifecycle of wireless sensor network nodes, provides a new opportunity for the development of energy-constrained networks, and has become one of the hot technologies currently researched in the field of wireless communication. SWIPT receivers convert collected RF signals into energy or information required by the terminal equipment.

When collecting wireless energy in the SWIPT network, the energy carried by the RF signal is transmitted to the receiving end through the antenna at the transmitting end. After collecting the RF signals, the antenna at the receiving end converts the energy in the signal through the nonlinear EH circuit and outputs the converted energy to the energy demand end to complete the collection, distribution, and utilization of wireless energy. The conversion efficiency of the RF to direct current (RF-DC) is a key indicator for evaluating the EH circuit, which indicates the ratio of the output power of the circuit to the power of the input RF signal. Many research works have shown that with the increase in the input RF signal power, the conversion efficiency of the RF-DC gradually decreases in the saturation region [9,10,11]. Therefore, in order to reduce the energy loss, designing new EH receivers and energy receiving mechanisms is important to improve the conversion efficiency of the RF-DC. Multiantenna technology can improve the channel capacity and communication rate. In the network based on SWIPT technology, multiple users can simultaneously transmit the required data in the same spectrum through multiantenna technology [12]. However, the combined application of the multiantenna technology and SWIPT technology not only has a large energy consumption [13], increasing the energy consumption costs, but also leads to serious inter-symbol interference and spectrum overlap. Therefore, it is not only necessary to design a communication system with high energy conversion efficiency to extend the lifecycle of energy-constrained terminal equipment but also to take effective measures to combat channel interference. It is worth noting that orthogonal frequency division multiplexing (OFDM) technology divides channels into mutually orthogonal subchannels, which can effectively reduce inter-symbol interference [14]. The combination of SWIPT and OFDM technologies can not only give full play to their respective technical advantages but can also effectively combat the above channel fading issues, significantly improving network throughput [15].

In addition, the broadcast characteristics of wireless channels can easily lead to user information leakage; so, it is an important issue to ensure the security of user communication. In [16], the author used the hybrid beamforming design scheme to improve the security energy efficiency of the system. In [17], the author used the signal-to-leakage-plus-noise ratio to improve the security energy efficiency of multibeam communication system. In [18], the author used the inter-user interference to realize the robust beamforming design, which aimed to enhance the system communication security and guarantee the EH requirements. When users collect wireless energy, other user information can be obtained through RF signals. To interfere with eavesdroppers intercepting legitimate user information, a common method is to add artificial noise (AN) to the received signal of the legitimate users [19,20].

In SWIPT-based networks, the application of multiantenna technology, AN technology, OFDM technology, etc., in network optimization and resource allocation has been studied in many existing works. In [15], based on the OFMA technology, in the IRS-assisted SWIPT network, the author used the nonlinear energy collection model to jointly optimize the PS ratio and transmit power to improve the network information transmission rate. In [21], a receiver based on double-layer power splitting (PS) architecture was used in a multiantenna SWIPT network to optimize the confidentiality energy efficiency. In [22], a multiuser SWIPT network model with nonlinear energy collection was constructed. In order to maximize the EH while minimizing the transmission power consumption, beamforming design and antenna selection schemes were layered and optimized. In [23], AN-assisted transmission was used to maximize the confidentiality efficiency of the SWIPT network and jointly optimized the digital precoding vectors, the AN covariance matrix, and power allocation ratios under nonlinear EH constraints. The authors in [21,24] studied the power allocation of multiuser multiple-input single-output (MISO) systems with multiple nonlinear EH circuit models, used multiple energy collection circuits to collect energy, and proposed PS EH receiver architecture, which breaks through the performance limitation of a single EH circuit on the system saturation effect. However, the distance of the PS EH system they designed was relatively complex, and there was still a certain distance from the actual application.

Improving the efficiency of wireless energy collection and the stability of energy supply can effectively extend the lifecycle of energy limited local area networks. Therefore, it is necessary to design a new type of EH model. With the explosive growth in the data transmission volume, it is necessary to improve the information transmission rate while ensuring communication security. Quantitative EH is the process of optimizing, segmenting, and collecting energy during the energy collection process. The nonlinear EH model can also adopt the method of quantifying the EH. The above research adopted the nonlinear EH model and did not study the information transmission rate of the secure SWIPT system based on the quantified EH model.

**Figure 1:**
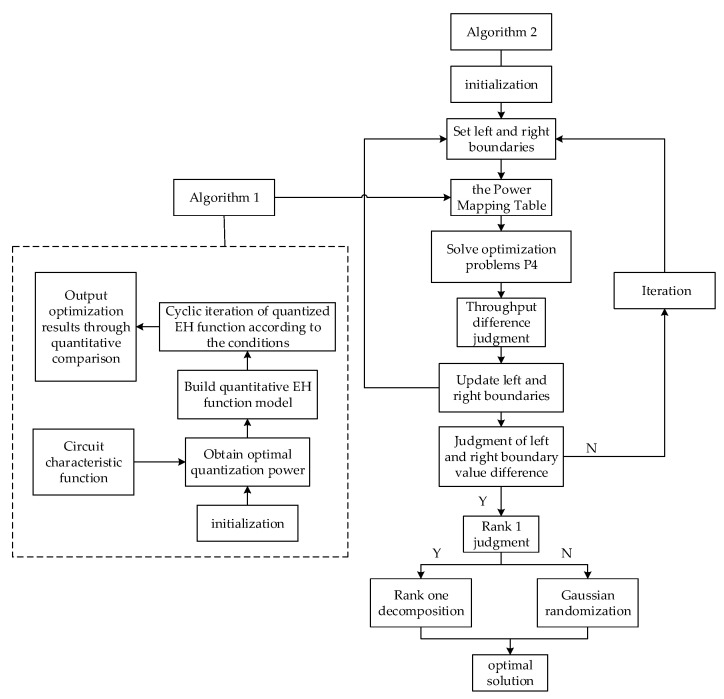
The flow charts of the proposed Algorithms 1 and 2.

Therefore, in order to fill the research gap, this paper explores the performance of a secure SWIPT network based on quantized EH receiver models. In this paper, we propose a quantitative EH model, which is applied to multiuser MISO-SWIPT networks to maximize the throughput of the SWIPT network, while meeting the signal-to-interference-plus-noise ratio (SINR) requirements of legitimate users, the EH requirements, the total transmission power of the base stations, and the security SINR threshold constraints. While ensuring the security of user communication, the transmission rate of information is improved. The main contributions are summarized as follows:

- We quantify the energy collection process to improve the conversion efficiency of the EH process. Compared with the receiver architecture containing multiple nonlinear EH circuits, the quantized power division receiver architecture proposed in this paper considers the RF-DC conversion efficiency of the circuit in the EH receiver, quantifies the input power of the receiver based on the optimal receiving power, and improves the conversion efficiency of the receiver EH process.
- To meet the user’s EH demand, an optimization algorithm (Figure 1) based on EH circuit characteristics is proposed to construct a power mapping table. In order to facilitate obtaining the minimum input power of the receiver corresponding to the user’s EH requirements, this paper proposes an optimization algorithm based on the circuit input and output power characteristics to determine the number of power shunts and constructs the corresponding power mapping table between the user’s EH demand and the minimum input power of the receiver. We search the power mapping table to find the circuit input power and power shunt number that meet the user’s EH needs.
- The hierarchical optimization method is used to solve the nonconvex optimization problem constructed in this paper. First, the equivalent transformation method is used to transform some constraints into convex constraints. Then, the power mapping table is used to transform the EH requirements constraints that are difficult to solve directly. Secondly, the variable replacement, semidefinite relaxation, binary optimization, and other methods are used to transform the problem into a convex optimization problem. Finally, the convex optimization problem is solved with the help of optimization tools CVX and the optimization algorithm.
- We design a joint optimization algorithm (Figure 1) based on a QPS receiver to maximize network throughput. With the help of the power mapping table, a joint optimization algorithm based on the quantized power segmentation and dichotomous method is designed to optimize the allocation of resources in the secure energy-carrying network based on QPS receivers and to maximize the throughput of SWIPT network.

The simulation results show that the joint optimization algorithm has good convergence under the quantized EH model. Compared with the PS receiver architecture, the QPS receiver architecture based on the nonlinear quantized power division model can avoid the EH circuit falling into the saturated working area and can maintain high network throughput. In addition, compared with the PS receiver architecture, the QPS receiver can maintain a near-optimal EH conversion efficiency in a wide receiving power domain, which helps to improve the RF-DC conversion efficiency of the receiver.

The rest of this paper is organized as follows. Section 2 presents the system and network models. In Section 3, the problem formulation, transformation methods, and optimization process are elaborated. In Section 4, the simulation results and analysis are provided. Finally, the paper is concluded in Section 5.
**Algorithm 1.** Optimize power allocation for the power mapping table1: Input parameters $a_{n}$, $b_{n}$ $M_{n}$, etc., $P_{n}(0)=0$;2: Let the input power $P_{n}(t)=t(mW)$, $f(t)=E_{n}^{l}/P_{n}^{l}$, l∈(0,1);3: $\eta_{Q}=P_{n}/E_{n}^{NL}$, $E_{n}^{NL}=E_{n}^{l}+[L]P_{o}$;4: $P_{o}=\arg\max f(t):=\{t|t>0\}$;5: $y_{n}(t/n)=E_{n}^{l}(t/n)=\frac{\varphi_{n}(P_{n}(t))-M_{n}\Omega_{n}}{1-\Omega_{n}}$;6: Loop solution;   initialization, t=0.1, T=70(mW), etc., shunt number $L_{o}=n=1$, $N\in Z^{+}$;   **for** t=0.1; t≤T; t=t+0.1 **do**     **if** n<=N, **then**      $y_{n}(t/n)>=y_{n+1}(t/n+1)$, output $y^{*}=y_{n}(t/n)$, $t^{*}=t$, $L_{o}^{*}=n^{*}=n$; **Break**;     **else** n=n+1; **return if**; 7: Output result, conversion efficiency: $\eta^{*}=y^{*}/t^{*}$; return $\left\{y^{*},t^{*},L_{o}^{*},\eta^{*},\eta_{Q}\right\}$.

**Algorithm 2.** The joint resource optimization algorithm based on the QPS EH mechanism and dichotomous method1: Initialization $\gamma_{n}$, $P_{\max}$, $\bar{\Gamma }$, K, $\delta_{n}^{2}$, N, and other parameters;2: Build the power mapping table through Algorithm 1;3: Input, l=0, r=1, t=0, C(0)=0, $\rho_{n}(t)=0$, and n=1,2,…,M;4: Let t=t+1, $\rho_{n}(t)=\frac{L+l}{2}$, n=1,2,…,M;5: Find the power mapping table according to the user requirements $E_{\min,n}$ to obtain $P_{n}$ and the optimal shunt number $L_{o}^{*}$;6: Solve the optimization problem $P_{4}$ to obtain $\left\{W_{n}^{*},W_{E}^{*},Z_{n}^{*}\right\}$ and C(t);7: **Update** $\Delta \rho_{n}(t)=\rho_{n}(t)+\tau$;8: **Substitute** $\Delta \rho_{n}(t)$ into $P_{4}$ to obtain ΔC(t);9: **If** ΔC(t)−C(t)>ξ, **then** $l=\rho_{n}(t)$, **else** $r=\rho_{n}(t)$;10: **Iteration** steps 4~10, **until** r−l≤ε;11: Assignment $\rho_{n}^{*}=l$, judge the rank of $W_{n}^{*}(t)$, $W_{E}^{*}(t)$, and $Z_{n}^{*}(t)$ separately; **if** the rank is 1, **then** perform rank-one decomposition, **else** Gaussian randomization is carried out to obtain the optimized variable, and obtain $w_{n}^{*}(t)$, $w_{E}^{*}(t)$, and $z_{n}^{*}(t)$;12: Return $\left\{w_{n}^{*},w_{E}^{*},z_{n}^{*},\rho_{n}^{*},L_{o}^{*}\right\}$.

## 2. System Model

### 2.1. Transmission Scheme

**Figure 2:**
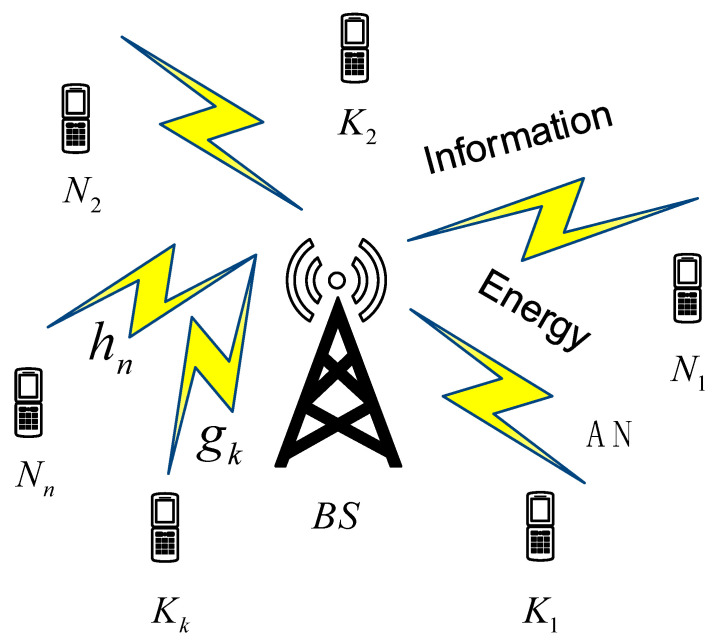
The multiuser MISO secure SWIPT network.

This paper investigates a multiuser MISO secure SWIPT network in the downlink. As shown in Figure 2, the SWIPT network adopts an OFDM access mode, there are I single antenna users within the coverage area of the multiantenna BS, and the network bandwidth is divided into multiple quadrature subchannels. When the BS transmits signals to N legitimate users with $N_{T}$ antennas, idle K users (K=I−N) may eavesdrop on legitimate user information. For simplicity, we use n and k to indicate the nth legitimate recipient and the kth eavesdropper, respectively, n∈N≜{1,2,…,N}, k∈K≜{1,2,…,K}.

In order to ensure the security of communication, artificial noise (AN) is embedded in the transmitted signal. Thus, the transmitted signal can be given by:

(1) $$x=\sum_{n=1}^{N}w_{n}s_{n}+z_{n}+w_{E},\;\forall n,$$

where $s_{n}\in \mathbb{C}$ represents the user’s independent Gaussian data symbol, $E\{|s_{n}{|}^{2}\}=1$, $w_{n}\in {\mathbb{C}}^{N_{T}\times 1}$ is the beamforming vector of the nth legitimate receiver, and $z_{n}\in {\mathbb{C}}^{N_{T}\times 1}$ represents the AN vector with a power of $P_{n}^{H}=Tr(z_{n}z_{n}^{H})$. $w_{E}$ is the energy signal transmitted by the transmitter, which is a Gaussian pseudo-random sequence that supplies energy to the user, i.e., $w_{E}∼CN\left(0,W_{E}\right)$, where $W_{E}$ represents the covariance matrix of the energy signal at the transmitter. In addition, all subchannels adopt block static flat fading channels, the channel vector within the block remains unchanged, and the equivalent channel responses from the BS to the nth legitimate user and the kth eavesdropping user are denoted by $h_{n}\in {\mathbb{C}}^{N_{T}\times 1}$ and $g_{k}\in {\mathbb{C}}^{N_{T}\times 1}$, respectively. The signal received by the nth legitimate user can be written as follows:

(2) $$y_{n}=h_{n}^{H}w_{n}s_{n}+h_{n}^{H}(z_{n}+w_{E})+n_{n},\;\forall n,$$

where $n_{n}∼CN\left(\sigma_{n}{}^{2}\right)$ is the Additive White Gaussian Noise (AWGN) at the nth user, and the power is $\sigma_{n}{}^{2}$. Since $\sigma_{n}{}^{2}$ generally has much lower noise power than the noise power $\delta_{n}^{2}$ introduced by the baseband processing circuit in ID process, similar to many existing works [21,25], $\sigma_{n}{}^{2}$ is ignored in the analysis below.

In the legal channel, the AN is transmitted in zero space and can be eliminated by the receiver. Let $z_{n}≜R_{n}d_{n}$, where $R_{n}R_{n}^{H}=I$, $d_{n}∼CN\left(0,\Sigma \right)$, $R_{n}$ is the orthogonal basis of $h_{n}^{H}$ zero space, and $h_{n}^{H}R_{n}=0$, so that the AN contained in the received signal by legitimate user n is completely eliminated. The receiving end of the legitimate user n applies PS power splitting technology, the power splitting ratio $\rho_{n}$ for ID, $0<\rho_{n}<1$, H, and the remaining power proportional $1-\rho_{n}$ is used for EH; so, the legitimate user n in the downlink receives the signal for EH, which can be expressed as:

(3) $$y_{n}^{EH}=\sqrt{1-\rho_{n}}(h_{n}^{H}w_{n}s_{n}+h_{n}^{H}w_{E}),\;\forall n.$$

Assuming that the interference with the energy signal $w_{E}$ can be perfectly eliminated by the ID process of each legitimate receiver [12], the signals collected for the ID by legitimate users can be given as:

(4) $$y_{n}^{ID}=\sqrt{\rho_{n}}(h_{n}^{H}w_{n}s_{n}+\vartheta_{n}),\;\forall n,$$

where $\vartheta_{n}∼CN\left(0,\delta_{n}^{2}\right)$ is the noise generated by the ID receiver processing the baseband signal, with a mean of zero and a variance of $\delta_{n}^{2}$. Since the OFDM access transmission mode is used, and the subchannels for transmitting information between users are orthogonal to each other, the SINR of the legitimate users n can be expressed as:

(5) $$SINR_{n}=\frac{\rho_{n}|h_{n}^{H}w_{n}{|}^{2}}{\delta_{n}^{2}},\;\forall n.$$

Eavesdropping users cannot obtain the AN signal designs for legitimate channels; so, the AN cannot be eliminated. When eavesdropping user k collects information from legitimate user n, its received signal can be given by:

(6) $$y_{k,n}^{EA}=g_{k}^{H}w_{n}s_{n}+g_{k}^{H}z_{n}+\vartheta_{n},\;\forall n.$$

The noise $\sigma_{n}^{2}$ generated by the antenna is small enough relative to the EH power to be ignored during the EH process [26]. According to the user’s EH power distribution ratio, the nth user’s EH power can be determined by:

(7) $$P_{n}=(1-\rho_{n})E\left\{\sum_{i=1}^{N}|h_{n}^{H}w_{n}{|}^{2}+|h_{n}^{H}w_{E}{|}^{2}+|h_{n}^{H}z_{n}{|}^{2}\right\},\;\forall n.$$

When user k intercepts the received information of legitimate user n, its SINR can be written as:

(8) $$y_{k,n}^{EA}(w_{n},z_{n})=\frac{|g_{k}^{H}w_{n}{|}^{2}}{|g_{k}^{H}w_{n}{|}^{2}+\delta_{n}^{2}},\;\forall n,k,$$

and the total transmit power of the BS can be given by:

(9) $$P_{T}(w_{n},w_{E},z_{n})=\sum_{n=1}^{N}(||w_{n}|{|}^{2}+||z_{n}|{|}^{2})+||w_{E}|{|}^{2},\;\forall n.$$

### 2.2. Quantitative EH Model

Due to the nonlinear characteristics of the diode, there is a saturated input voltage. Therefore, in the nonlinear EH circuit, during the process of gradually increasing the input power, the RF-DC conversion efficiency of the actual energy acquisition circuit first increases and then decreases, and the energy conversion efficiency is not high. Thus, when the input power is large, it is necessary to use the multi-energy harvesting circuit to collect energy, and the energy allocation problem of the multi-energy harvesting circuit is worth studying.

**Figure 3:**
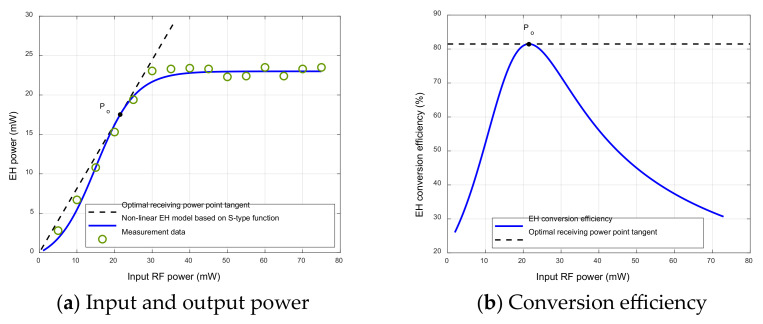
The input and output power figure and the conversion efficiency figure of the single EH circuit. (**a**) A comparison between the output power for the S-type function EH model in [27] and the measurement data from practical EH circuits in [28]; (**b**) the conversion efficiency figure of the single EH circuit.

From a practical perspective, this section introduces a nonlinear EH model with adjustable parameters based on the sigmoidal (S-type) function [27], which combines the actual measurement data of [28] and adjusts the parameters to accurately simulate the input and output power characteristics of the EH circuits. The EH model [27], based on the S-type function has been widely used in the nonlinear energy collection network model [24,26,29,30] since it was proposed. The input and output power characteristics and conversion efficiency curves of the EH circuit are shown in Figure 3. It is worth noting that when the input power is connected to the area around 21.5 mW, it is closer to the saturation output power (23 mW) of the EH circuit, and the conversion efficiency is higher. In this case, while maintaining high conversion efficiency, it ensures the efficient utilization of the input power threshold of the EH circuit. Based on the above analysis and experimental comparison of the EH circuit’s power distribution with a nonlinear S-type function EH model, a receiver system architecture using quantified power allocation is proposed.

In the ID, in order to avoid the input power of the EH circuit in the saturation zone, a quantified power-splitting (QPS) receiver based on multiple EH circuits is proposed. Since the ID and EH processes are in different modules, the QPS receiver divides the RF signal power received by legitimate user n into two parts, one for the ID and one for the EH. Then, according to the characteristics of the circuit, as shown in Figure 3a, since the ratio of the input power to the output power of a single EH circuit is maximum at $P_{o}$, the received power $P_{n}$ is quantized by the input power at $P_{o}$ (i.e., quantization power), and the number of power shunts is determined by the ratio of the received power to the quantified power, which is given by the following formula:

(10) $$L=\frac{P_{n}}{P_{o}},\;\forall n,$$

(11) l=L−[L],

(12) $$P_{n}{}^{l}=lP_{n},\;\forall n.$$

Let $L_{o}=[L]$, represent the number of power shunts, [·] represents the rounding operation, and l is the fractional part of L. For a legitimate user n, the total power collected during the EH process in the QPS receiver can be expressed as:

(13) $$E_{n}^{NL}=E_{n}^{l}+[L]P_{o},\;\forall n,$$

(14) $$E_{n}^{l}=\frac{\varphi_{n}-M_{n}\Omega_{n}}{1-\Omega_{n}},\;\forall n,$$

(15) $$\Omega_{n}=\frac{1}{1+\exp(a_{n}b_{n})},\;\forall n,$$

(16) $$\varphi_{n}=\frac{M_{n}}{1+\exp(-a_{n}(P_{n}{}^{l}-b_{n}))},\;\forall n,$$

where $\varphi_{n}$ is the sigmoidal function, and $\Omega_{n}$ is a constant to guarantee an input/output response of zero. In addition, parameters $a_{n}$ and $b_{n}$ are constants related to circuit characteristics. $M_{n}$ is a constant representing the maximum output power of a single EH circuit at saturation. Note that parameters $a_{n}$, $b_{n}$, and $M_{n}$ can be obtained by the curve fitting tool [27]. The proposed QSP EH model is used to describe the throughput of the system, and the resource allocation algorithm is designed. The network throughput based on the QPS receiver architecture can be given by $C=T\overset{N}{\underset{n=1}{\sum }}\log\left(1+\frac{\rho_{n}|h_{n}^{H}w_{n}{|}^{2}}{\delta_{n}^{2}}\right)$.

## 3. Problem Formulation and Transformation Solution

### 3.1. Problem Formulation

In the multiantenna MISO-SWIPT communication network based on OFDM, the nonlinear multi-circuit QPS EH model is used to establish the optimization problem $P_{1}$, with the constraints of the legitimate users’ SINR requirements, the EH requirements, the total transmission power of BS, and the security SINR threshold, aiming at maximizing the throughput of the SWIPT network. This optimization problem is mathematically formulated as:

(17) $$P_{1}:\;\underset{\left|w_{n},w_{E},z_{n},\rho_{n},L_{o}\right|}{\max}T\overset{N}{\underset{n=1}{\sum }}\log\left(1+\frac{\rho_{n}|h_{n}^{H}w_{n}{|}^{2}}{\delta_{n}^{2}}\right)$$

(18) $$\mathrm{subject}\;\mathrm{to}\;\frac{\rho_{n}|h_{n}^{H}w_{n}{|}^{2}}{\delta_{n}^{2}}\geq \gamma_{n},\;\forall n,$$

(19) $$\overset{N}{\underset{n=1}{\sum }}\left(||w_{n}|{|}^{2}+||z_{n}|{|}^{2}\right)+Tr\left(W_{E}\right)⩽P_{\max}$$

(20) $$E_{n}^{NL}\geq E_{\min,n},\;\forall n,$$

(21) $$\frac{|g_{k}w_{n}{|}^{2}}{|g_{k}w_{n}{|}^{2}+\delta_{n}^{2}}\leq \bar{\Gamma },\;\forall n,k,$$

(22) $$0<\rho_{n}<1,\;\forall n,$$

(23) $$W_{E}≻=0,$$

(24) $$L_{o}\in N^{+}.$$

In the formula, $\gamma_{n}$, $E_{\min,n}$, and $\bar{\Gamma }$, respectively indicate for the target SINR, the minimum harvest power required by user n, the maximum transmission power of the BS, and the upper limit of the SINR noise ratio of the illegally received signal by the eavesdropping user; $L_{o}$ is a positive integer, and (23) is the semi-positive definite Hermitian matrix of the energy signal. However, the constraint set in problem $P_{1}$ contains quadratic terms, has variable coupling, and is a nonconvex constraint set, which makes the problem difficult to solve.

### 3.2. Problem Transformation

#### 3.2.1. Constraints Transformation

To solve problem $P_{1}$, the partial constraint is expressed in an equivalent form. First, the nonconvex constraints (18) and (20) of problem $P_{1}$ are expressed in equivalent form, i.e., the requirement for the signal quality and the requirements for the EH. Equivalently, we express constraint (18) as:

(25) $$\frac{|h_{k}^{H}w_{k}{|}^{2}}{\gamma_{k}}\geq \frac{\delta_{n}^{2}}{\rho_{n}},\;\forall n.$$

Since $1/\rho_{n}$ is a convex function, (25) is a convex constraint on $\rho_{n}$. Note that because $\Omega_{n}$ has no effect on the design of the optimization problem, $E_{n}^{NL}$ is used directly to describe the harvested power at the nth user. Thus, the inverse function of (15) can be written as:

(26) $$P_{n}\left(E_{n}^{l}\right)=b_{n}-\frac{1}{a_{n}}\ln\left(\frac{M_{n}-E_{n}^{l}}{E_{n}^{l}}\right),\;\forall n,$$

and with Equations (10), (13), and (26), constraints (20) can be transformed as:

(27) $$\sum_{n=1}^{N}\left({\left|h_{n}^{H}w_{n}\right|}^{2}+{\left|h_{n}^{H}z_{n}\right|}^{2}\right)+{\left|h_{n}^{H}w_{E}\right|}^{2}\geq \frac{P_{n}\left(E_{\min,n}\right)}{1-\rho_{n}},\;\forall n,$$

which is a convex constraint about $\rho_{n}$, because $1/(1-\rho_{n})$ is a convex function. Inequality (21) can be replaced with the following constraints:

(28) $${\left|g_{n}^{H}w_{n}\right|}^{2}-\bar{\Gamma }{\left|g_{n}^{H}w_{n}\right|}^{2}\leq \delta_{n}^{2},\;\forall n.$$

The following determines the more independent integer variable $L_{o}$. When the EH circuit characteristics are determined, for a given EH requirement $E_{\min,n}$, each power shunt number $L_{o}$ has a unique optimal EH circuit number corresponding to it. Based on this observation, by searching for the conversion efficiency mapping table corresponding to the EH circuit’s input and output power, the uniquely determined optimal number of power shunts can be obtained. Part of the conversion efficiency mapping is shown in Table 1, and the method of obtaining Table 1 is summarized as Algorithm 1.

#### 3.2.2. The Power Mapping Table

The steps of applying Algorithm 1 to construct the power mapping table are summarized on page 4.

Algorithm 1 starts from the actual characteristics of the EH circuit; when determining the number of power shunts, the number of comparisons is small, which is effective for determining the $L_{o}$ from small to large, and the mapping table required for EH can be constructed efficiently and completely. In [24], the author adopted the SMO method (with a computational complexity of O(M^2L)), and the proposed Algorithm 1 has a lower computational complexity (O(10NT)).

In addition, building the mapping table offline does not add computational complexity to the inline beamforming design. Table 1 shows an example of a mapping table, where $M_{k}=23\;\mathrm{mW}$, and $L_{o}^{*}$ represents the optimal number of power shunts. $\eta_{2}$, $\eta_{3}$, and $\eta^{*}$ represent the two and three equal divisions of power $P_{n}$ and the optimal conversion efficiency, respectively.

The conversion efficiency units are %, the power units are mW, $\eta_{Q}$, and $\eta_{1}$, which represent the RF-DC conversion efficiency of the QPS receivers and the traditional single-circuit EH receivers, respectively, and $\eta_{1}P_{n}<E_{\min,n}$ indicates that the user EH requirements are not supported. It can be seen that the QPS receiver improves the conversion efficiency of the RF-DC and, more importantly, breaks through the limitation of the output DC power. According to the correspondence of the mapping table, the EH requirements constraint of user n can be satisfied by the convex constraint (27).

**Figure 4:**
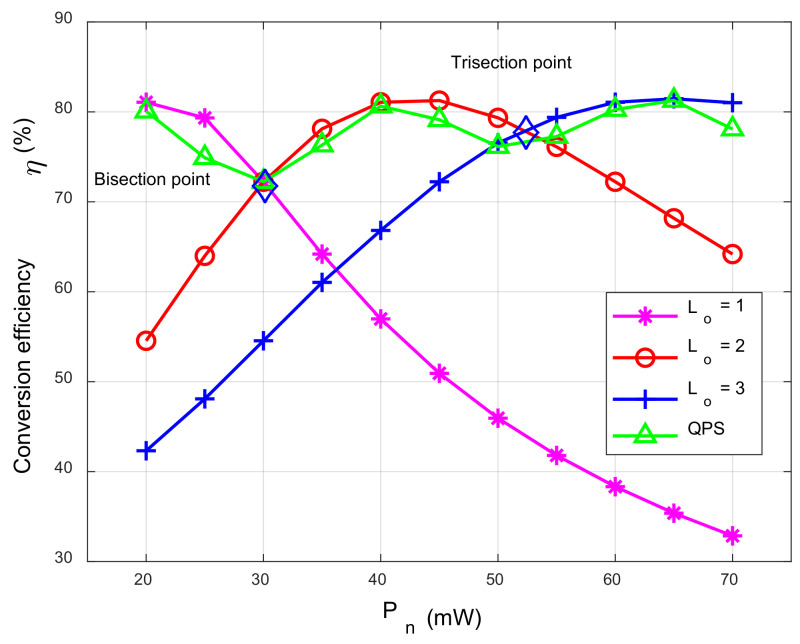
The partial conversion efficiency comparison curve.

Figure 4 shows the conversion efficiency trend plotted by the reference Algorithm 1, showing the change trend of the input and output conversion efficiency of some EH circuits. With the gradual increase in the input power, when the number of circuits is 1~3, only part of the power segment occupies the optimal EH conversion efficiency; in sharp contrast to this, with the increase in the input power, the conversion efficiency ($\eta_{Q}$) of the quantified power input curve was around the optimal EH conversion efficiency, fluctuating slightly and tending toward the optimal EH conversion efficiency. It can be seen that the quantized power input scheme has better stability than the optimal shunt input scheme, and the EH conversion efficiency $\eta_{Q}$ of the QPS receiver tends to the optimal EH conversion efficiency when the input power is large. Therefore, a quantized QPS receiver with stable output EH conversion efficiency is adopted.

#### 3.2.3. Variable Substitution and Optimization Solution

We define $Z_{n}=z_{n}z_{n}^{H},\forall n$ and obtain $||w_{n}|{|}^{2}=Tr\left(W_{n}\right)$, $||z_{n}|{|}^{2}=Tr\left(Z_{n}\right)$; similarly, we define $H_{n}=h_{n}h_{n}^{H}$ and $G_{k}=g_{k}g_{k}^{H}$,∀n,k, the matrix $W_{n}$ is positively definite, satisfying $Rank(W_{n})=1,\forall n$, and after variable substitution, the optimization problem $P_{1}$ can be rewritten as:

(29) $$P_{2}:\;\underset{\left|W_{n},W_{E},Z_{n},\rho_{n}\right|}{\max}T\overset{N}{\underset{n=1}{\sum }}\log\left(1+\frac{\rho_{n}Tr\left(H_{n}W_{n}\right)}{\delta_{n}^{2}}\right).$$

(30) $$s.t.\;\frac{Tr\left(H_{n}W_{n}\right)}{\gamma_{n}}\geq \frac{\delta_{n}^{2}}{\rho_{n}},\;\forall n,$$

(31) $$\overset{N}{\underset{n=1}{\sum }}\left(Tr\left(W_{n}\right)+Tr\left(Z_{n}\right)\right)+Tr\left(W_{E}\right)⩽P_{\max},$$

(32) $$\overset{N}{\underset{n=1}{\sum }}\left(Tr\left(H_{n}W_{n}\right)+Tr\left(H_{n}Z_{n}\right)\right)+Tr\left(H_{n}W_{E}\right)\geq \frac{P_{n}\left(E_{\min,n}\right)}{1-\rho_{n}},\;\forall n,$$

(33) $$Tr\left(G_{k}W_{n}\right)-\bar{\Gamma }Tr\left(G_{k}Z_{n}\right)⩽\delta_{n}^{2},\;\forall n,k,$$

(34) $$0<\rho_{n}<1,\;\forall n,$$

(35) $$W_{n},W_{E},Z_{n}≻=0,\;\forall n,$$

(36) $$Rank(W_{n})=1,Rank(W_{E})=1,Rank(Z_{n})=1,\;\forall n.$$

Using the semidefinite relaxation (SDR) method, dropping the rank-one constraint in the problem $P_{2}$, the optimization problem can be relaxed as:

(37) $$P_{3}:\;\underset{\left|W_{n},W_{E},Z_{n},\rho_{n}\right|}{\max}T\overset{N}{\underset{n=1}{\sum }}\log\left(1+\frac{\rho_{n}Tr\left(H_{n}W_{n}\right)}{\delta_{n}^{2}}\right),$$

(38) s.t. (30)−(35).

Since the objective function (29) and constraints (30) and (32) have coupling variables $\rho_{n}$, when $\rho_{n}$ is determined, the optimization problem $P_{3}$ can be rewritten as:

(39) $$P_{4}:\;\underset{\left|W_{n},W_{E},Z_{n}\right|}{\max}T\overset{N}{\underset{n=1}{\sum }}\log\left(1+\frac{\rho_{n}Tr\left(H_{n}W_{n}\right)}{\delta_{n}^{2}}\right),$$

(40) s.t. (30)−(35).

As shown in Equation (41), the Hessen matrix of the objective function of problem $P_{4}$ is $\nabla_{\alpha }^{2}$ and has eigenvalues less than zero; thus, Equation (39) is a concave function. Constraints (30)–(35) are convex sets. Therefore, the optimization problem $P_{4}$ is a convex problem that can be solved by the CVX toolbox [31].

(41) $$\nabla_{\alpha }^{2}=\left[\begin{array}{cc} \frac{-1}{{\left(\frac{\delta_{n}^{2}}{\rho_{n}^{2}}+Tr(H_{n}W_{n})\right)}^{2}} & 0\\ 0 & 0 \end{array}\right].$$

For the variable $\rho_{n}$, within its feasible range, the optimal solution $\rho_{n}^{*}$ can be obtained by dichotomy, and after determining $\rho_{n}^{*}$, the optimization tool CVX is used to solve the problem $P_{4}$ to obtain the optimal solution {$W_{n}^{*},W_{E}^{*},Z_{n}^{*}$}. The $P_{1}$ problem is equivalent to the $P_{2}$ problem, the $P_{2}$ problem is transformed into the $P_{3}$ problem by the SDR method, and the solution to the $P_{3}$ problem is an approximate solution of $P_{2}$. Thus, the solution to the $P_{3}$ problem, which is obtained by using the dichotomy and the CVX tool, is the approximate optimal solution of the original optimization problem $P_{1}$.

The solution method of the original problem is summarized as an optimization Algorithm 2, which is based on the QPS EH mechanism and the dichotomous method, and is summarized on page 5.

## 4. Simulation Results

This section compares and analyzes the performance of the joint optimization algorithm under the EH models and QSP receiver architecture. In the multiuser MISO secure SWIPT network model, as shown in Figure 2, the distance between the BS and the legitimate users as well as the eavesdropping users was equal, and both were set as d=2 m. The number of antennas equipped with the BS was $N_{T}=4$. The legitimate users and the eavesdropping users were equipped with a single antenna. The target SINR of the legitimate user was $\gamma_{n}=10\mathrm{dB}$. In addition, the BS transmit power was $P_{\max}=\bar{P}\times N=30\mathrm{dBm}$. The number of legitimate users was N=3, and the number of eavesdropping users was K=1. The noise power at the ID process was $\delta_{n}^{2}=-40\mathrm{dBm}$. Channel models $h_{n}$ and $g_{k}$ are random variables that follow the Gaussian distribution of zero mean and unit variance. The user’s EH threshold $E_{\min,n}=-42\mathrm{mW}$, and the security SINR threshold $\bar{\Gamma }=0\mathrm{dB}$. The parameters of the nonlinear EH circuits were $M_{n}=23\mathrm{mW}$, a=1900 and, b=0.003.

**Figure 5:**
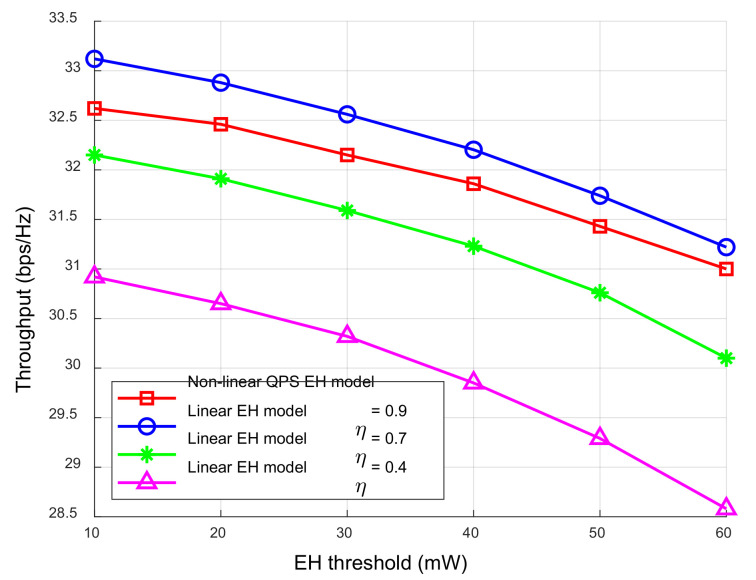
The relationship between the throughput and the EH threshold under different EH models.

When the transmission power of the BS was fixed, the relationship between the system throughput and the EH threshold under different EH models is shown in Figure 5. The system throughput decreased with the increase in the EH threshold. Since the BS transmission power remained unchanged, more power was allocated to the EH process. The energy allocated to the ID process was reduced, and the system data transmission rate was reduced. Compared with the steeper curve of the linear EH model, the trend of the nonlinear QPS EH model curve was more realistic. When the conversion efficiency of the linear EH model was set to 0.4 and 0.7, the energy that could be collected by the EH circuit was reduced due to misestimation, which could not meet the user’s energy collection needs; thus, the algorithm designed in this paper allocates more energy to the EH process under the linear EH model. When the linear EH conversion efficiency reached 0.9, as the energy collection circuit reached the saturation zone, the conversion efficiency decreased, and the actual collected energy was less than the theoretical calculation, resulting in the user’s EH requirements not being met. Therefore, compared with the linear EH model, the resource allocation scheme based on the nonlinear EH model can predict the network performance more accurately and meet the user’s EH requirements.

**Figure 6:**
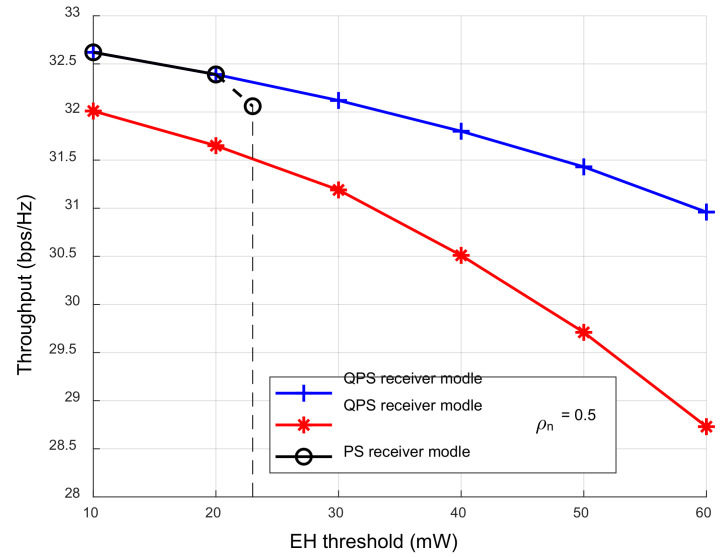
The throughput under different receiver architectures varies with the EH threshold.

Figure 6 shows the variation trend of the network throughput with user EH thresholds under different receiver architectures. Compared with other receiver models, the QPS receiver model maintained a high network throughput. With the increase in the EH threshold, the energy used to decode the legitimate user information decreased; thus, the system throughput under different receiver models gradually decreased. When the single PS receiver model was adopted, and the EH threshold was greater than 20 mW, the EH capacity range of the PS receiver exceeded the EH threshold and could not meet the user’s EH requirements. When the EH threshold was greater than 23 mW, due to the limitation of the saturated output power (the saturation output power threshold was 23 mW), the system allocated all the energy to the EH process, which still could not meet the user’s EH requirements, and the system could not perform the ID process. Although the limitation of saturated output power could be avoided by using a QPS receiver model with a power splitting ratio of 0.5, the reachable network throughput was lower than that of the QPS receiver model, because the receive power splitting ratio $\rho_{n}$ was not optimized.

**Figure 7:**
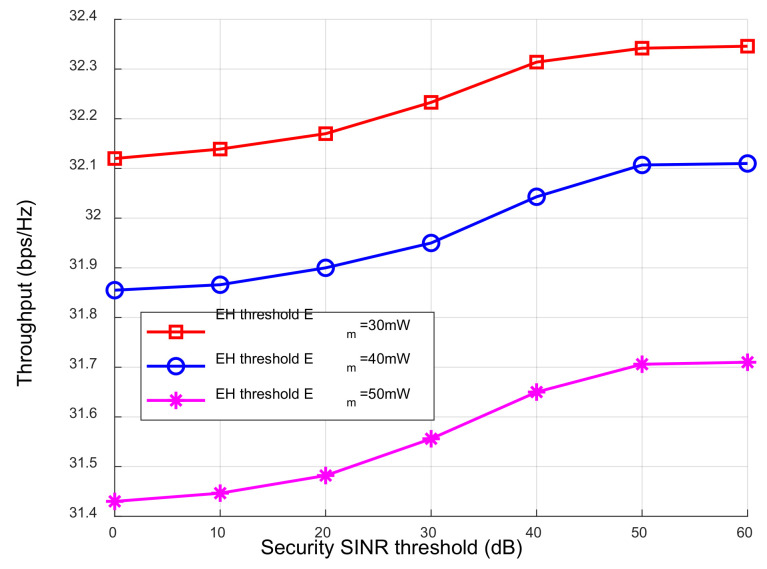
The network throughput under different security thresholds.

The relationship between the system throughput between the different EH thresholds and the safety SINR thresholds is shown in Figure 7. The system throughput increased with the increase in the safety SINR threshold. The reason is that when the security SINR threshold of eavesdropping users increased, the power allocated to the AN decreased, and the energy used for the ID processing of legitimate users increased, resulting in greater system throughput. When the security SINR was constant, the system throughput decreased with the increase in the EH threshold. The reason is that the energy used for the ID decreased with the increase in the user’s EH requirements, resulting in the decrease in the information transmission rate.

**Figure 8:**
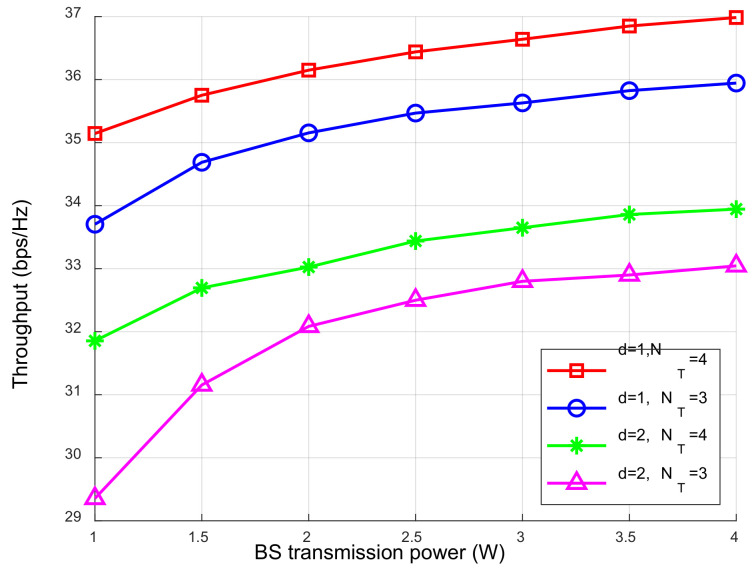
The relationship between the network throughput and BS transmit power.

The relationship between different BS transmission powers and network throughputs is shown in Figure 8. The network throughput increased with the total transmission power of the BS. This is because when the network EH threshold and security SINR remained unchanged, the energy used for the ID increased with the increase in the BS total transmission power, resulting in greater network throughput. When the transmission distance was constant, the network throughput increased with the increase in the number of the BS antennas. The reason is that the channel gain of SWIPT system increased with the increase in the number of the BS antennas, and the transmission quality of the signal increased. When the number of the BS antennas was constant, the network throughput decreased with the increase in the distance between the BS and the user. The reason is that with the increase in the transmission distance between the user and the BS, the signal transmission path loss increased, which made the channel transmission quality worse, resulting in a decrease in network throughput.

**Figure 9:**
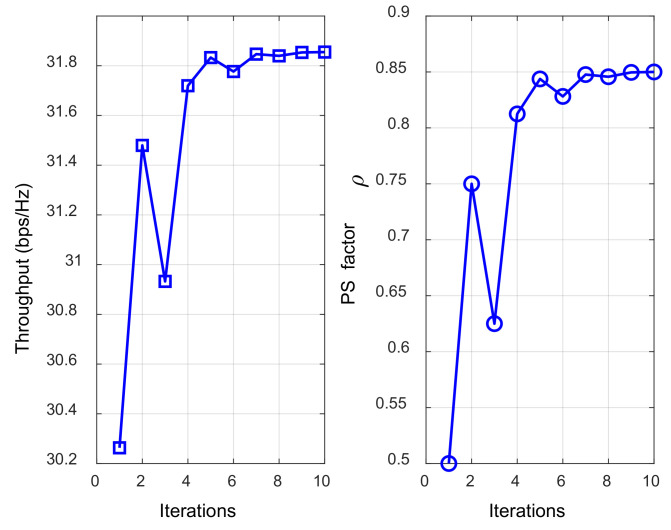
The convergence effect of the joint optimization algorithms.

The convergence of the joint optimization algorithms based on the quantized EH and dichotomous method is shown in Figure 9. Due to the advantages of the dichotomous method and the range of the PS factor being (0, 1), in the first five iterations of the network throughput and the PS factor, the search range of the optimization variables of the algorithm was rapidly narrowed, and the range of the optimal value was approximated. Then, the algorithm began to refine the search, converged at the tenth iteration, and the PS factor and network throughput reached the optimal value, indicating that the joint optimization algorithm had good convergence.

## 5. Conclusions

This paper aimed to maximize the network throughput and study the resource allocation problem in multiuser MISO secure SWIPT networks with an AN. Inspired by the nonlinear EH model, a QSP receiver architecture based on quantitative EH mechanism was designed, and the receiver was applied to the EH process to avoid the receiver’s EH circuit operating in the saturation region. Under the conditions of satisfying the requirements of legitimate users’ SINR, the EH requirements, the total transmitted power of the BS, and the security SINR threshold constraints, a joint optimization algorithm based on the quantization model was proposed to solve the nonconvex optimization problem of maximizing the network throughput. The QSP receiver architecture proposed in this paper can achieve near optimal EH conversion efficiency, and compared with the PS receiver architecture, the input power threshold range is larger, which can avoid the saturation zone of the EH circuit and maintain high network throughput. However, the QPS EH mechanism proposed in this paper has not been applied in practice. Therefore, the gap between the theoretical and practical applications provides a direction for future research.

## Figures and Tables

**Table 1:** Part of the power mapping table ($M_{n}=23\;\mathrm{mW}$).

$P_{n}$	$E_{\min,n}$	$\eta_{1}$	$\eta_{2}$	$\eta_{3}$	$\eta_{Q}$	η*	$L_{o}^{*}$
29	20.91	73.83	70.74	/	72.09	73.83	1
31	22.52	70.61	73.62	/	72.64	73.62	2
35	26.71	64.19	78.12	61.04	76.31	78.12	2
40	32.26	56.98	81.07	66.82	80.64	81.07	2
50	38.07	45.94	79.34	76.54	76.14	79.34	2
52	39.60	44.19	78.16	77.75	76.15	78.16	3
53	40.49	43.36	77.51	78.47	76.40	78.47	3
55	42.49	41.80	76.11	79.39	77.25	79.39	3
58	45.87	39.64	73.83	80.54	79.09	80.54	3
60	48.16	38.33	72.23	81.07	80.27	81.07	3

## Data Availability

Data are contained within the article.

## References

[1] Lv Z., Lou R., Li J., Singh A.K., Song H. (2021). Big data analytics for 6G-enabled massive internet of things. IEEE Internet Things J..

[2] Liu J.S., Lin C.H.R., Hu Y.C., Donta P.K. (2022). Joint beamforming, power allocation, and splitting control for SWIPT-enabled IoT networks with deep reinforcement learning and game theory. Sensors.

[3] Chettri L., Bera R. (2019). A Comprehensive Survey on Internet of Things (IoT) Toward 5G Wireless Systems. IEEE Internet Things J..

[4] Tin P.T., Nguyen T.N., Tran D.H., Voznak M., Phan V.-D., Chatzinotas S. (2021). Performance enhancement for full-duplex relaying with time-switching-based SWIPT in wireless sensors networks. Sensors.

[5] Ijemaru G.K., Ang K.L.M., Seng J.K.P. (2022). Wireless power transfer and energy harvesting in distributed sensor networks: Survey, opportunities, and challenges. Int. J. Distrib. Sens. Netw..

[6] Huang J., Zhou Y., Ning Z., Gharavi H. (2019). Wireless power transfer and energy harvesting: Current status and future prospects. IEEE Wirel. Commun..

[7] Basim M., Khan D., Ain Q.U., Shehzad K., Shah S.A.A., Jang B.-G., Pu Y.-G., Yoo J.-M., Kim J.-T., Lee K.-Y. (2022). A Highly Efficient RF-DC Converter for Energy Harvesting Applications Using a Threshold Voltage Cancellation Scheme. Sensors.

[8] Perera T.D.P., Jayakody D.N.K., Pitas I., Garg S. (2020). Age of information in swipt-enabled wireless communication system for 5GB. IEEE Wirel. Commun..

[9] Boshkovska E., Ng D.W.K., Zlatanov N., Koelpin A., Schober R. (2017). Robust resource allocation for MIMO wireless powered communication networks based on a non-linear EH model. IEEE Trans. Commun..

[10] Huda S.M.A., Arafat M.Y., Moh S. (2022). Wireless power transfer in wirelessly powered sensor networks: A review of recent progress. Sensors.

[11] Xiong K., Wang B., Liu K.J.R. (2017). Rate-energy region of SWIPT for MIMO broadcasting under nonlinear energy harvesting model. IEEE Trans. Wirel. Commun..

[12] Ng D.W.K., Lo E.S., Schober R. (2014). Robust beamforming for secure communication in systems with wireless information and power transfer. IEEE Trans. Wirel. Commun..

[13] Ai B., Guan K., He R., Li J., Li G., He D., Zhong Z., Huq K.M.S. (2017). On indoor millimeter wave massive MIMO channels: Measurement and simulation. IEEE J. Select. Areas Commun..

[14] Hwang T., Yang C., Wu G., Li S., Li G.Y. (2008). OFDM and its wireless applications: A survey. IEEE Trans. Veh. Technol..

[15] Peng X., Wu P., Tan H., Xia M. (2022). Optimization for IRS-Assisted MIMO-OFDM SWIPT System with Nonlinear EH Model. IEEE Internet Things J..

[16] Lin Z., Lin M., Champagne B., Zhu W.-P., Al-Dhahir N. (2021). Secrecy-energy efficient hybrid beamforming for satellite-terrestrial integrated networks. IEEE Trans. Commun..

[17] Lin Z., An K., Niu H., Hu Y., Chatzinotas S., Zheng G., Wang J. (2023). SLNR-based secure energy efficient beamforming in multibeam satellite systems. IEEE Trans. Aerosp. Electron. Syst..

[18] Lin Z., Lin M., Zhu W.P., Wang J.-B., Cheng J. (2020). Robust secure beamforming for wireless powered cognitive satellite-terrestrial networks. IEEE Trans. Cogn. Commun. Netw..

[19] Phan V.D., Nguyen T.N., Le A.V., Voznak M. (2021). A study of physical layer security in SWIPT-based decode-and-forward relay networks with dynamic power splitting. Sensors.

[20] Xu Y., Xie H., Liang C., Yu F.R. (2021). Robust secure energy-efficiency optimization in SWIPT-aided heterogeneous networks with a nonlinear energy-harvesting model. IEEE Internet Things J..

[21] Lu Y., Xiong K., Fan P., Ding Z., Zhong Z., Ben Letaief K. (2020). Secrecy energy efficiency in multi-antenna SWIPT networks with dual-layer PS receivers. IEEE Trans. Wirel. Commun..

[22] Jalali J., Khalili A., Rezaei A., Famaey J., Saad W. Power-efficient Antenna Switching and Beamforming Design for Multiuser SWIPT with Non-Linear Energy Harvesting. Proceedings of the 2023 IEEE 20th Consumer Communications & Networking Conference (CCNC).

[23] Zhu Z., Ma M., Sun G., Hao W., Liu P., Chu Z., Lee I. (2022). Secrecy Rate Optimization in Nonlinear Energy Harvesting Model-Based mmWave IoT Systems With SWIPT. IEEE Syst. J..

[24] Lu Y., Xiong K., Fan P., Zhong Z., Ai B., Letaief K.B. (2021). Worst-case energy efficiency in secure SWIPT networks with rate-splitting ID and power-splitting EH receivers. IEEE Trans. Wirel. Commun..

[25] Chen H., Li Y., Jiang Y., Ma Y., Vucetic B. (2014). Distributed power splitting for SWIPT in relay interference channels using game theory. IEEE Trans. Wirel. Commun..

[26] Zargari S., Khalili A., Wu Q., Mili M.R., Ng D.W.K. (2021). Max-min fair energy-efficient beamforming design for intelligent reflecting surface-aided SWIPT systems with non-linear energy harvesting model. IEEE Trans. Veh. Technol..

[27] Boshkovska E., Ng D.W.K., Zlatanov N., Schober R. (2015). Practical non-linear energy harvesting model and resource allocation for SWIPT systems. IEEE Commun. Lett..

[28] Guo J., Zhu X. An improved analytical model for RF-DC conversion efficiency in microwave rectifiers. Proceedings of the 2012 IEEE/MTT-S International Microwave Symposium Digest.

[29] Peng C., Chen Y., Chen Q., Tang Z., Li L., Gui W. (2021). A remaining useful life prognosis of turbofan engine using temporal and spatial feature fusion. Sensors.

[30] Gautam S., Solanki S., Sharma S.K., Chatzinotas S., Ottersten B. (2021). Hybrid active-and-passive relaying model for 6G-IoT greencom networks with SWIPT. Sensors.

[31] Boyd S., Boyd S.P., Vandenberghe L. (2004). Convex Optimization.

